# Predicting Public Uptake of Digital Contact Tracing During the COVID-19 Pandemic: Results From a Nationwide Survey in Singapore

**DOI:** 10.2196/24730

**Published:** 2021-02-03

**Authors:** Young Ern Saw, Edina Yi-Qin Tan, Jessica Shijia Liu, Jean CJ Liu

**Affiliations:** 1 Division of Social Sciences Yale-NUS College Singapore Singapore; 2 Neuroscience and Behavioral Disorders Programme Duke-NUS Medical School Singapore Singapore

**Keywords:** contact tracing, COVID-19, mobile app, digital health, epidemiology

## Abstract

**Background:**

During the COVID-19 pandemic, new digital solutions have been developed for infection control. In particular, contact tracing mobile apps provide a means for governments to manage both health and economic concerns. However, public reception of these apps is paramount to their success, and global uptake rates have been low.

**Objective:**

In this study, we sought to identify the characteristics of individuals or factors potentially associated with voluntary downloads of a contact tracing mobile app in Singapore.

**Methods:**

A cohort of 505 adults from the general community completed an online survey. As the primary outcome measure, participants were asked to indicate whether they had downloaded the contact tracing app TraceTogether introduced at the national level. The following were assessed as predictor variables: (1) participant demographics, (2) behavioral modifications on account of the pandemic, and (3) pandemic severity (the number of cases and lockdown status).

**Results:**

Within our data set, the strongest predictor of the uptake of TraceTogether was the extent to which individuals had already adjusted their lifestyles because of the pandemic (z=13.56; *P*<.001). Network analyses revealed that uptake was most related to the following: using hand sanitizers, avoiding public transport, and preferring outdoor over indoor venues during the pandemic. However, demographic and situational characteristics were not significantly associated with app downloads.

**Conclusions:**

Efforts to introduce contact tracing apps could capitalize on pandemic-related behavioral adjustments among individuals. Given that a large number of individuals is required to download contact tracing apps for contact tracing to be effective, further studies are required to understand how citizens respond to contact tracing apps.

**Trial Registration:**

ClinicalTrials.gov NCT04468581, https://clinicaltrials.gov/ct2/show/NCT04468581

## Introduction

### Background

In May 2020, Google and Apple released the Exposure Notification System, which is an application programming interface that logs the following: who a phone user has been in contact with, for how long, and at what distance [1]. This release came 2 months after COVID-19 was declared a pandemic [2], allowing governments to identify and isolate contacts of confirmed cases through a process known as “contact tracing” [3,4].

Less than a year after the first reported cases, over 33 million individuals have tested positive for COVID-19 worldwide and more than 1 million have died [5]. To limit disease spread, over half of the global population has been subjected to lockdowns involving school closures, workplace shutdowns, and movement restrictions [6]. Although these lockdowns are effective in tapering the epidemic curve [7], they are costly to the global economy and are unsustainable [8]. However, allowing the virus to spread unhindered could overwhelm the health care system and result in large-scale mortality [9,10].

To address both infection control and economic concerns, several countries have turned to contact tracing to keep the economy running [11,12]. Epidemiological modeling suggests that if (1) cases are effectively identified (through rigorous testing protocols), (2) contact tracing is comprehensive (identifying all possible exposure), and (3) contacts are quarantined in a timely manner, this strategy can curb the spread of the virus [4,11]. In an optimal scenario, 80% of contacts should be traced on the same day an individual tests positive [3,11].

### Conventional Versus Digital Contact Tracing

Early during the pandemic (and in previous infectious disease outbreaks), contact tracing was manually performed [13]. Using a range of interview and surveillance techniques, a human contact tracer would typically identify an average of 36 contacts for each positive case [14]. Although this strategy allows for high levels of case detection when there are few cases [15], its labor-intensive format—requiring ~12 h of tracing for each positive case [16]—is difficult to scale up. Additionally, individuals who test positive may forget whom they have been in contact with, thus undermining the effectiveness of the process [11].

Considering these limitations of manual contact tracing, several mobile apps have been developed to facilitate automated contact tracing [17], for example, COVID Watch in the United States [18], COVIDSafe in Australia [19], and Corona-Warn-App in Germany [20]. These apps primarily track Bluetooth signals from phones in the vicinity [3], capturing contacts without the restraints of staffing or recall biases [4,11]. Further, phone apps can notify individuals swiftly after a contact tests positive, allowing them to be quickly isolated [3].

### Understanding the Predictors of Uptake

Despite the potential of digital contact tracing, a recent meta-analysis concluded that owing to implementation barriers, manual contact tracing should remain the order of the day [12]. One major barrier pertains to the uptake of mobile apps. Several modelling studies have assessed parameters needed for the COVID-19 reproduction number (*R_0_*) to fall below 1 [3,11,21]. *R_0_* refers to the number of infections spread from 1 positive case, and a value less than 1 indicates that the virus has been contained. For this to be achieved, contact tracing apps need to be downloaded by at least 56% of the population [21], which is much higher than the average rate of downloads globally (9%) [22].

To increase uptake, Qatar made it mandatory for residents to use the official contact tracing app [23]. Although this legislation led to high download rates (>90% [24]), the potential backlash from the public (eg, because of privacy concerns [25,26]) implies that few countries are likely to follow suit. Correspondingly, public health agencies would benefit from an understanding of the predictors of voluntary downloads [27], providing an empirical basis to nudge citizens and residents to voluntarily download contact tracing apps [28].

### The Current Study

Given the urgent need to boost contact tracing apps, this study is the first to identify sociodemographic factors predicting voluntary uptake. Our study was conducted in Singapore, where the world’s first nationwide contact tracing app TraceTogether was launched in March 2020 [29]. TraceTogether uses a centralized approach adopted by several governments [19]; namely, randomly generated user IDs are generated and shared via Bluetooth with phones in close proximity [30]. When individuals test positive for COVID-19, they consent to add both their own user IDs and those of their contacts to a centralized database. This is used to identify matches, and exposure notifications are then sent from the server to close contacts [31,32]. (As an alternative model, a decentralized approach could be used where both matches and notifications are made through the user’s phone [33].)

As Singapore was the forerunner of this technology, the app has accrued 2.3 million users within 6 months, including approximately 40% of Singapore’s resident population or 50% of all smartphone users (considering a smartphone penetration rate of 82%) [34,35]. Correspondingly, our study represents a “best case scenario” for app uptake after several months have elapsed. In terms of the epidemic curve, our study was conducted between April and July 2020, as the country was in a lockdown (April to May 2020). This period witnessed a peak in daily COVID-19 cases (April: >1000/day or 175 per million population), which gradually tapered over time (July: >100/day or 17.5 per million population).

## Methods

### Study Design and Population

Between April 3 and July 17, 2020, we recruited 505 adults who met the following eligibility criteria: (1) at least 21 years of age and (2) had lived in Singapore for a minimum of 2 years. All participants responded to online advertisements. Within the constraints of online sampling owing to the pandemic, we strove to obtain a representative sample by placing advertisements in a wide range of online community groups (eg, Facebook or WhatsApp groups among individuals in residential estates, universities, and workplaces) and by using paid online advertisements targeting the broad spectrum of Singapore residents.

Prior to study enrolment, participants provided informed consent in accordance with a protocol approved by the Yale-NUS College Ethics Review Committee (#2020-CERC-001; ClinicalTrials.gov ID NCT04468581). They then completed a 10-min online survey hosted on the platform Qualtrics [36]. Data were collected in accordance with the second phase of a larger study tracking COVID-19 responses, and findings from the first phase have been described previously [37,38].

### Outcome Variable: Use of TraceTogether

As the primary outcome variable, participants were asked to indicate whether they had downloaded the government’s contact tracing app TraceTogether (binary variables: 1=they had, 0=they had not).

### Predictors

#### Demographics and Situational Variables

As predictors of TraceTogether usage, participants then reported the following demographic data: age, gender, citizenship, ethnicity, marital status, education level, house type (a proxy of socioeconomic status in Singapore), and household size. Based on the survey timestamp, we also included the following as predictors: (1) the total number of cases in Singapore to date, (2) whether the nation was in a lockdown at the time of participation (0=no, 1=yes), and (3) a self-reported measure of confidence the government could control COVID-19 spread (4-point scale: 1=“not confident at all,” 4=“very confident”).

#### Other Behavioral Modifications

As a basis of comparison, participants were also asked to identify which of 18 other behavioral modifications they had made as a result of the pandemic (apart from downloading TraceTogether). Specifically, participants were asked whether they had (1) washed their hands more frequently, (2) used hand sanitizers, (3) worn a mask in public voluntarily (before a law was passed), (4) avoided taking public transport, (5) stayed home more than usual, (6) avoided crowded places, (7) chosen outdoor over indoor venues, (8) missed or postponed social events, (9) changed their travel plans voluntarily, (10) reduced physical contact with others (eg, by not shaking hands), (11) avoided visiting hospitals or other health care settings, (12) avoided visiting places where COVID-19 cases had been reported, (13) maintained distance from people suspected of recent contact with a COVID-19–positive individual, (14) maintained distance from people who might have recently traveled to countries with an outbreak, (15) maintained distance from people with flu-like symptoms, (16) relied more on online shopping (eg, for groceries), (17) stocked up on more household supplies and groceries than usual, or (18) taken their children out of school (for each item, 0=the measure was not taken, 1=the measure was taken). These values were then summed to compute an aggregated measure of behavioral change (out of 18), and were included as a predictor to assess whether contact tracing usage was associated with conventional behavioral modifications one undertakes during an epidemic [39,40].

As part of the survey, participants were also asked to specify any other behavioral modifications (n=9, 1.8%) or no other behavioral modification (n=2, 0.4%). However, these data were excluded from the statistical analyses owing to the low base rate of affirmative responses.

### Data Analysis Plan

For primary analysis, binary logistic regression was used to identify predictors TraceTogether uptake. In the first model (model 1), participants' demographics were included as predictors (age, citizenship, gender, marital status, education level, ethnicity, household type, and household size). Citizenship (base=others), gender (base=female), marital status (base=single), and ethnicity (base=Chinese) were coded as dummy variables. In the second model (model 2), we repeated the first model with the inclusion of situational variables (log-transformed total number of COVID-19 cases to date and lockdown status). Finally, in the third model (model 3), we repeated the second model with the inclusion of the total number of behavioral modifications as a predictor. All data were analyzed using SPSS (version 23, IBM Corp) and R (version 3.6.0, The R Foundation), with the type 1 familywise error rate controlled at α=.05 via Bonferroni correction (Bonferroni-adjusted α=.003 [.05/17 predictors]).

## Results

### Demographics of the Sample

Table 1 shows the wide range of demographic characteristics of our study cohort (N=505). Compared to the resident population, the sample was matched in the following characteristics: ethnic composition, household size, and housing type (a proxy of socioeconomic status in Singapore) (≤10% difference). However, compared to the resident population, the present participants were more likely to be female (n=313, 62.0% vs 51.1%), single or dating (n=234, 46.4% vs 31.6%), to have a higher level of education or no tertiary education (n=65, 12.9% vs 51.7%), and to be citizens of Singapore or of other countries (n=456, 90.3% vs 61.4%).

**Table 1:** Baseline characteristics of survey respondents (N=505).

Variable		Value
Age (years), mean (SD)		37.82 (11.31)
Number of behavioral modifications, mean (SD)		9.81 (3.82)
**Gender, n (%)**		
	Female	313 (62.0)
	Male	192 (38.0)
**Citizenship, n (%)**		
	Singaporean	456 (90.3)
	Others	49 (9.7)
**Highest education, n (%)**		
	No formal education	2 (0.4)
	Primary school	2 (0.4)
	Secondary school	23 (4.6)
	Junior college	26 (5.1)
	Institution of Technical Education	12 (2.4)
	Polytechnic (diploma)	88 (17.4)
	University (degree)	265 (52.5)
	Postgraduate (masters/PhD)	87 (17.2)
**Ethnicity, n (%)**		
	Chinese	412 (81.6)
	Malay	38 (7.5)
	Indian	32 (6.3)
	Eurasian	15 (3.0)
	Others	8 (1.6)
**Marital status, n (%)**		
	Single	170 (33.7)
	Dating	64 (12.7)
	Married	241 (47.7)
	Widowed/separated/divorced	30 (5.9)
**Household type, n (%)**		
	HDB^a^ flat: 1-2 rooms	14 (2.8)
	HDB flat: 3 rooms	50 (9.9)
	HDB flat: 4 rooms	132 (26.1)
	HDB flat: 5 rooms or executive flats	149 (29.5)
	Condominium or private apartments	122 (24.2)
	Landed property	38 (7.5)
**Household size, n (%)**		
	1	26 (5.1)
	2	88 (17.4)
	3	119 (23.6)
	4	133 (26.3)
	5+	139 (27.5)

^a^HDB: Housing & Development Board.

### Binary Logistic Regression

Of the 505 participants, 274 (54.3%; 95% CI 49.8%-58.7%) reported having downloaded TraceTogether. The download rate in this sample matches that of smartphone users in the resident population [34], and Table 2 describes the characteristics of users and nonusers.

Table 3 shows parameter estimates from logistic regression analyses of the predictors of TraceTogether uptake. No demographic or situational variable significantly predicted downloads (models 1 and 2). After controlling for these variables, the number of behavioral modifications emerged as a significant predictor (model 3); that is, with each unit increase in the number of behavioral modifications adopted, participants were 1.10 times more likely to download TraceTogether (*z*=13.56; *P*<.001).

**Table 2:** Characteristics of the users of TraceTogether (N=505).

Variable		TraceTogether usage	
		Users (n=274)	Nonusers (n=231)
Age (years), mean (SD)		38.57 (11.57)	36.95 (10.96)
Household type, mean (SD)		3.92 (1.18)	3.76 (1.21)
Household size, mean (SD)		3.54 (1.26)	3.53 (1.15)
Number of behavioral modifications, mean (SD)		10.33 (3.83)	8.96 (3.65)
**Gender, n (%)**			
	Female	177 (64.6)	136 (58.9)
	Male	97 (35.4)	95 (41.1)
**Citizenship, n (%)**			
	Singaporean	240 (87.6)	216 (93.5)
	Others	34 (12.4)	15 (6.5)
**Highest education, n (%)**			
	No formal education	1 (0.4)	1 (0.4)
	Primary school	1 (0.4)	1 (0.4)
	Secondary school	15 (5.5)	8 (3.5)
	Junior college	14 (5.1)	12 (5.2)
	Institution of Technical Education	8 (2.9)	4 (1.7)
	Polytechnic (diploma)	41 (15.0)	47 (20.3)
	University (degree)	136 (49.6)	129 (55.8)
	Postgraduate (masters/PhD)	58 (21.2)	29 (12.6)
**Ethnicity, n (%)**			
	Chinese	218 (79.6)	194 (84.0)
	Malay	20 (7.3)	18 (7.8)
	Indian	19 (6.9)	13 (5.6)
	Eurasian	13 (4.7)	2 (0.9)
	Others	4 (1.5)	4 (1.7)
**Marital status, n (%)**			
	Single	85 (31.0)	85 (36.8)
	Dating	38 (13.9)	26 (11.3)
	Married	133 (48.5)	108 (46.8)
	Widowed/separated/divorced	18 (6.6)	12 (5.2)

**Table 3:** Logistic regression models of predictors of the uptake of TraceTogether (dependent variable=downloaded TraceTogether).

Variable		Model 1: demographics^a^		Model 2: demographics and situational variables		Model 3: demographics, situational variables, and behavioral modifications	
		Odds ratio (95% CI)	*P* value	Odds ratio (95% CI)	*P* value	Odds ratio (95% CI)	*P* value
Age (years)		1.018 (1.00-1.04)	.09	1.020 (1.00-1.04)	.06	1.021 (1.00-1.04)	.05
Gender (base=female)		0.771 (0.53-1.12)	.17	0.799 (0.55-1.17)	.25	0.904 (0.61-1.34)	.61
Citizenship (base=others)		0.546 (0.26-1.14)	.11	0.597 (0.28-1.13)	.17	0.651 (0.30-1.39)	.27
Household type		1.082 (0.92-1.27)	.76	1.056 (0.90-1.25)	.52	1.011 (0.85-1.20)	.90
Household size		1.042 (0.89-1.23)	.62	1.028 (0.87-1.21)	.74	1.036 (0.88-1.22)	.67
Highest education		1.032 (0.89-1.19)	.67	1.029 (0.89-1.25)	.70	0.993 (0.85-1.16)	.92
**Ethnicity (base=Chinese)**							
	Malay	1.057 (0.53-2.11)	.88	1.050 (0.52-2.14)	.89	0.980 (0.48-2.02)	.96
	Indian	1.112 (0.52-2.37)	.78	0.984 (0.45-2.13)	.97	0.928 (0.43-2.03)	.85
	Eurasian	3.454 (0.70-17.02)	.13	3.475 (0.70-17.37)	.13	3.402 (0.66-17.42)	.14
	Others	0.720 (0.16-3.17)	.66	0.724 (0.16-3.29)	.68	0.851 (0.18-4.037)	.84
**Marital status (base=single)**							
	Dating	1.505 (0.82-2.76)	.19	1.555 (0.84-2.90)	.16	1.392 (0.74-2.63)	.31
	Married	0.968 (0.62-1.52)	.89	0.974 (0.61-1.55)	.91	0.900 (0.56-1.44)	.66
	Widowed/separated/divorced	1.146 (0.47-2.79)	.76	1.155 (0.47-2.84)	.75	1.034 (0.41-2.59)	.94
Local COVID-19 cases to date (log)		N/A^b^	N/A	0.774 (0.50-1.21)	.26	0.752 (0.48-1.18)	.22
Lockdown (base=no lockdown)		N/A	N/A	0.561 (0.30-1.04)	.07	0.599 (0.32-1.12)	.11
Confidence in the government		N/A	N/A	1.372 (1.05-1.79)	.02	1.363 (1.04-1.79)	.03
Number of behavioral modifications		N/A	N/A	N/A	N/A	1.102 (1.05-1.16)	<.001^c^

^a^Model 1: Overall percentage of users correctly classified-56.6%, Nagelkerke R2-0.048; Model 2: Overall percentage of users correctly classified-58.2%, Nagelkerke R2-0.068; Model 3: Overall percentage of users correctly classified-60.2%, Nagelkerke R2-0.103.

^b^N/A: not applicable.

^c^*P*<.003 (following Bonferroni corrections).

### Post Hoc Network Analysis

In the logistic regression analyses, TraceTogether downloads were predicted from the number of behavioral modifications because of the pandemic. To understand this association better, we conducted further exploratory analyses.

**Figure 1:**
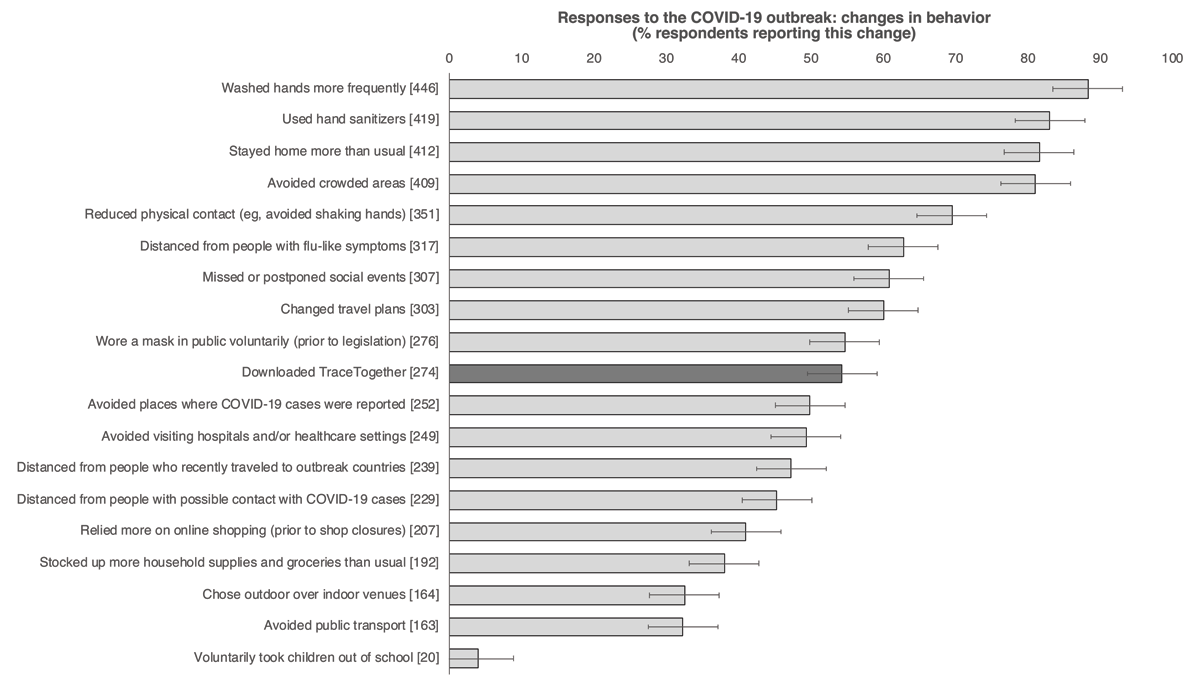
Self-reported behavioral modifications , other than downloading TraceTogether, among the study participants undertaken in response to the COVID-19 outbreak in Singapore. Error bars=95% CI. Numbers in brackets represent the total number of respondents who reported the behavioral change.

As shown in Figure 1, the majority of participants had modified their behaviors to curb the spread of COVID-19. The use of TraceTogether ranked 10th in the frequency of adoption (274/505, 54.3%), similar to the frequency of voluntary mask wearing (n=276, 54.7%).

A corollary question is how TraceTogether usage is associated with other health protective behaviors; that is, how likely were people to download TraceTogether if they had modified their behavior in other ways? To address this question, we conducted network analyses by estimating a mixed graphical model (MGM) with the R package *mgm* [41]. MGM constructs weighted and undirected networks where the pathways among behaviors represent conditionally dependent associations, having controlled for the other associations in the network. Similar to partial correlations, each association (or “edge”) is the average regression coefficient of two nodes. To avoid false-positive findings, we set small associations to 0 for the main models.

**Figure 2:**
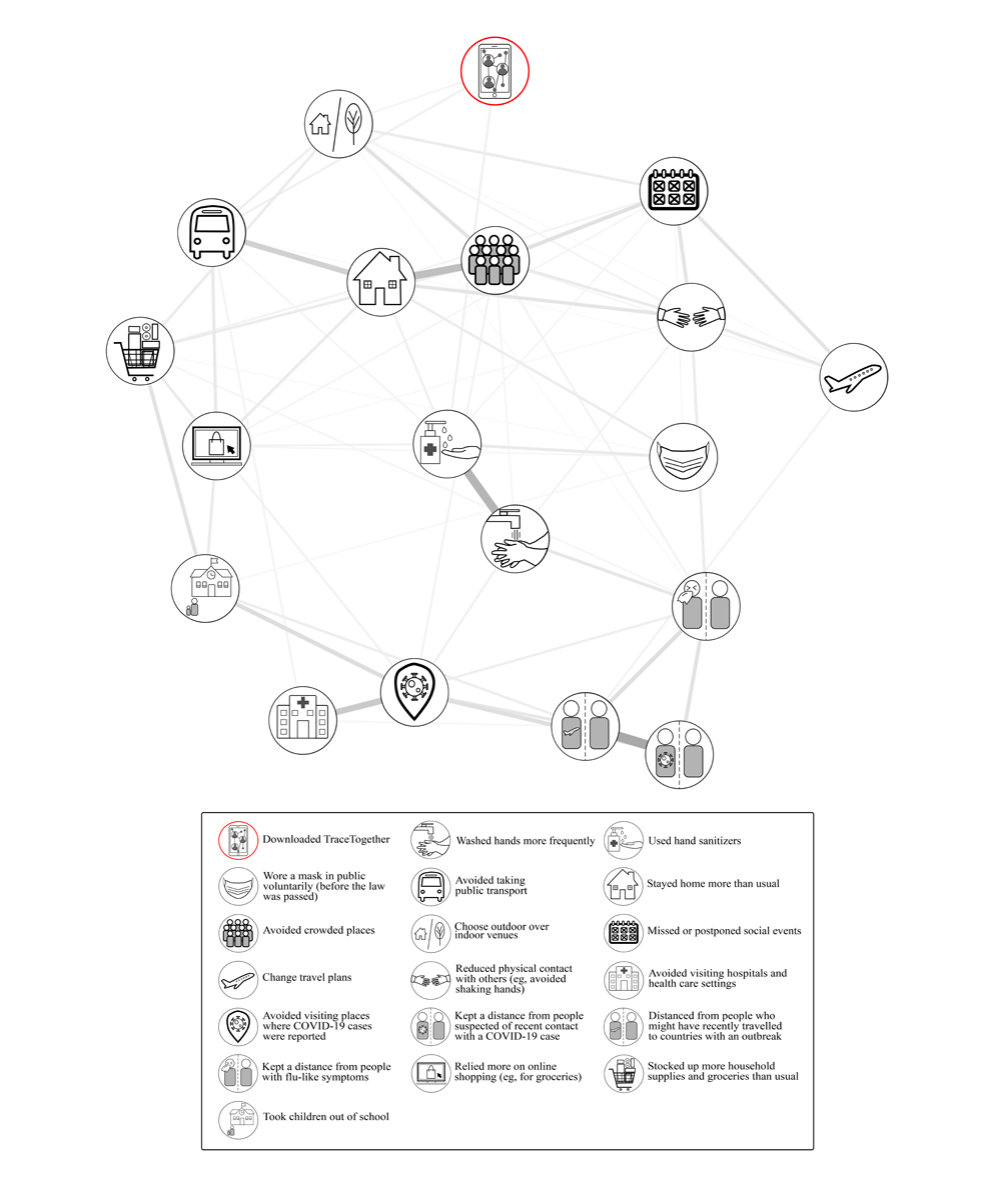
A model depicting how TraceTogether usage relates to other pandemic-related behavioral changes. Line thickness represents the strength of an association.

As shown in Figure 2, TraceTogether usage was associated with hand sanitizer use, avoidance of public transport, and a preference for outdoor vs indoor venues. The adjacency matrix (ie, numerical values for the average regression coefficient between two nodes) for Figure 2 is presented in Multimedia Appendix 1.

For sensitivity analysis, we performed logistic regression analysis using TraceTogether downloads as the dependent variable, and 18 other behavioral modifications (see Methods) as the predictors. Our conclusions did not change, as indicated in Multimedia Appendix 2.

## Discussion

As lockdowns owing to COVID-19 ease globally, digital contact tracing will play an increasingly critical role in managing the epidemic curve. However, this requires the public to actively download a contact tracing app—a step that has proven elusive among public health agencies worldwide [12]. This study is the first to examine the demographic, behavioral, and situational factors potentially predicting the voluntary use of TraceTogether.

### Behavioral Modifications

As our primary outcome, we observed that the number of behavioral modifications significantly predicted the use of TraceTogether. In other words, a person who had already changed his/her lifestyle on account of the pandemic was also likely to download a contact tracing app. Network analyses revealed that downloads clustered with (1) using hand sanitizers, (2) avoiding public transport, and (3) preferring outdoor to indoor venues. This finding may suggest that public health campaigns could capitalize on other behavioral modifications when seeking to promote app downloads, for example, by printing information regarding a contact tracing app on the packaging of hand sanitizers or by framing the use of digital contact tracing as a preventive behavior. Policy makers might also expect app download rates to track behavioral modifications, anticipating, for example, higher download rates when the public fears an increase in COVID-19 cases (leading to more behavioral modifications) [42].

Theoretically, our findings further corroborate those of previous studies on how individuals change their behaviors during a pandemic. Based on prior outbreaks, a taxonomy of modifications had been identified whereby (1) “avoidant behaviors” are measures taken to avoid contact with potential carriers (eg, avoiding crowded places), while (2) “prevention behaviors” are those associated with maintaining hygiene (eg, regular hand washing) [42]. Extrapolating to the technological realm, our findings suggest that the use of a contact tracing app cuts across this taxonomy, since downloads were associated with *both* avoidant (avoiding public transport and preferring outdoor venues) and prevention behaviors (using hand sanitizers). Moving forward, we urge further studies to revise these classification systems in light of new technological developments.

### Demographic and Situational Factors

Apart from behavioral modifications, it is notable that no demographic (eg, age, gender, etc) or situational variable (eg, number of COVID-19 cases and lockdown status) significantly predicted TraceTogether uptake. Prior to our study, it would have been conceivable that only a subset of the population would download a contact tracing app (eg, demographic groups based on gender, educational level, or age) [27,38,43]. By contrast, our findings highlight how uptake of digital contact tracing apps cuts across demographic groups.

While the lack of significant associations may be counterintuitive, a recent study reported similar results when predicting COVID-19–related behavioral modifications [42]. In a multinational survey, Harper et al [42] similarly observed that demographic and situational variables were unrelated to behavioral modifications owing to the pandemic. Since behavioral modifications predicted the use of a digital contact tracing app in our study, it seems reasonable to observe an analogous pattern here; that is, our results are unlikely to be based on false-negative outcomes.

As public health agencies develop strategies to promote downloads for contact tracing apps, the pattern of our findings may in turn suggest that demographic-specific messages are not needed. This is encouraging because the behavioral sciences offer widespread measures to “nudge” the general population [44]. In this case, the general public simply needs a one-off nudge to download the contact tracing app, after which the app functions independently in the background. Thus, if governments can nudge users in this first step (eg, by introducing incentives to download or by introducing contact tracing as an opt-out feature of existing government apps), it may be possible to attain the download rates necessary for contact tracing to be effective. Simultaneously, we urge further studies on the acceptance of such strategies; considering public concerns regarding privacy [25,26], any widespread intervention would need to be introduced cautiously.

### Limitations

Our study has several limitations of note. As the first study of its kind, we made several choices at the exclusion of others. First, we opted for a cross-sectional design that precludes strong conclusions regarding causality. Second, we included an online sample to minimize person-to-person contact during the pandemic. Although we sampled individuals from a wide array of demographic groups, respondents were not representative of the general nationwide population; this may have deterred the establishment of potential associations among variables (eg, by including a high proportion of educated participants). Third, our survey relied on participants’ self-reported use of a contact tracing app. Although our download rate is similar to that of the general population, further studies may seek to verify actual usage (eg, by incorporating survey questions in a contact tracing app). Fourth, our survey was not intended to measure every aspect of TraceTogether usage, and there were several notable omissions (eg, reasons why individuals chose to use or not use the app, phone ownership, and usage-related questions). Indeed, our model metrics (eg, small Nagelkerke R^2^) indicate small effect sizes, highlighting the need for further studies to include a more comprehensive set of variables that may account for app downloads. Finally, we examined TraceTogether—an app with a centralized contact tracing protocol. Future studies are required to assess whether our findings extend to apps with decentralized protocols or to other forms of digital contact tracing that do not rely on mobile apps (eg, public acceptance of cloud-based contact in South Korea).

### Conclusion

In conclusion, the potential contribution of digital technology to pandemic management is receiving increasing attention. What remains unclear, however, is how this technology is received and how best to promote its uptake. Focusing on contact tracing, this study shows that downloads of a mobile app was best predicted from the adoption of other infection control measures such as increased hand hygiene. In other words, the introduction of digital contact tracing is not merely a call to “trace together” but rather to “modify together,” to use contact tracing apps as part of the broader spectrum of behavioral modifications during a pandemic.

## Appendix

Multimedia Appendix 1 Adjacency matrix.

Multimedia Appendix 2 Sensitivity analysis.

## Abbreviations

MGM: mixed graphical model
R_0_: reproduction number
