# Supplementary material for: Predicting Public Uptake of Digital Contact Tracing During the COVID-19 Pandemic: Results From a Nationwide Survey in Singapore
Source: J Med Internet Res. 2021 Feb 3;23(2):e24730. doi: 10.2196/24730 (PMC7861036; doi:10.2196/24730)
Supplement: Multimedia Appendix 1 [file jmir_v23i2e24730_app1.pdf]

Appendix A

S1. Adjacency Matrix

	1	2	3	4	5	6	7	8	9	10	11	12	13	14	15	16	17	18	19
1	0.00	1.05	0.00	0.00	0.00	0.00	0.13	0.00	0.00	0.00	0.16	0.00	0.20	0.00	0.00	0.34	0.00	0.00	0.00
2	1.05	0.00	0.30	0.20	0.14	0.21	0.00	0.00	0.13	0.00	0.00	0.00	0.00	0.00	0.00	0.00	0.18	0.00	0.00
3	0.00	0.30	0.00	0.00	0.00	0.21	0.00	0.00	0.10	0.00	0.00	0.00	0.00	0.00	0.00	0.00	0.12	0.08	0.10
4	0.00	0.20	0.00	0.00	0.20	0.00	0.00	0.14	0.00	0.00	0.00	0.00	0.00	0.00	0.00	0.00	0.00	0.00	0.00
5	0.00	0.14	0.00	0.20	0.00	0.65	0.00	0.22	0.00	0.07	0.00	0.16	0.00	0.00	0.00	0.00	0.31	0.00	0.00
6	0.00	0.21	0.21	0.00	0.65	0.00	0.91	0.00	0.15	0.00	0.36	0.00	0.00	0.00	0.00	0.00	0.28	0.18	0.00
7	0.13	0.00	0.00	0.00	0.00	0.91	0.00	0.40	0.42	0.12	0.29	0.00	0.17	0.00	0.00	0.17	0.00	0.27	0.00
8	0.00	0.00	0.00	0.14	0.22	0.00	0.40	0.00	0.26	0.06	0.15	0.00	0.00	0.00	0.00	0.08	0.00	0.28	0.00
9	0.00	0.13	0.10	0.00	0.00	0.15	0.42	0.26	0.00	0.38	0.38	0.00	0.00	0.00	0.00	0.00	0.13	0.00	0.00
10	0.00	0.00	0.00	0.00	0.07	0.00	0.12	0.06	0.38	0.00	0.33	0.00	0.00	0.00	0.16	0.00	0.00	0.00	0.00
11	0.16	0.00	0.00	0.00	0.00	0.36	0.29	0.15	0.38	0.33	0.00	0.00	0.00	0.00	0.00	0.27	0.08	0.00	0.00
12	0.00	0.00	0.00	0.00	0.16	0.00	0.00	0.00	0.00	0.00	0.00	0.00	0.75	0.00	0.45	0.34	0.00	0.00	0.00
13	0.20	0.00	0.00	0.00	0.00	0.00	0.17	0.00	0.00	0.00	0.00	0.75	0.00	0.45	0.34	0.00	0.19	0.00	0.48
14	0.00	0.00	0.00	0.00	0.00	0.00	0.00	0.00	0.00	0.00	0.00	0.00	0.45	0.00	1.22	0.41	0.00	0.00	0.00
15	0.00	0.00	0.00	0.00	0.00	0.00	0.00	0.00	0.00	0.16	0.00	0.45	0.34	1.22	0.00	0.46	0.00	0.00	0.25
16	0.34	0.00	0.00	0.00	0.00	0.00	0.17	0.08	0.00	0.00	0.27	0.34	0.00	0.41	0.46	0.00	0.00	0.13	0.00
17	0.00	0.18	0.12	0.00	0.31	0.28	0.00	0.00	0.13	0.00	0.08	0.00	0.19	0.00	0.00	0.00	0.00	0.28	0.28
18	0.00	0.00	0.08	0.00	0.00	0.18	0.27	0.28	0.00	0.00	0.00	0.00	0.00	0.00	0.00	0.13	0.28	0.00	0.41
19	0.00	0.00	0.10	0.00	0.00	0.00	0.00	0.00	0.00	0.00	0.00	0.00	0.48	0.00	0.25	0.00	0.28	0.41	0.00

1	Washed my hands more frequently	11	Reduced physical contact with others (e.g., avoided shaking hands)
2	Used hand sanitisers	12	Avoided visiting hospitals and/or healthcare settings
3	Wore a mask in public voluntarily (before the law was passed)	13	Avoided visiting places where COVID-19 cases were reported
4	Downloaded the government's TraceTogether application	14	Kept a distance from people suspected of recent contact with a COVID-19 case
5	Avoided taking public transport	15	Kept a distance from people who might have recently travelled to countries with an outbreak
6	Stayed home more than usual	16	Kept a distance from people with flu symptoms
7	Avoided crowded places	17	Relied more on online shopping (e.g., for groceries)
8	Choose outdoor over indoor venues	18	Stored up more household and/or food supplies than usual
9	Missed or postponed social events	19	Took children out of school

Table S1. Adjacency Matrix of the 19 Behavioral Modification
