# Supplementary material for: Predicting Public Uptake of Digital Contact Tracing During the COVID-19 Pandemic: Results From a Nationwide Survey in Singapore
Source: J Med Internet Res. 2021 Feb 3;23(2):e24730. doi: 10.2196/24730 (PMC7861036; doi:10.2196/24730)
Supplement: Multimedia Appendix 2 [file jmir_v23i2e24730_app2.pdf]

Appendix B

S2. Sensitivity Analysis

Dependent Variable: Downloaded the Contact Tracing Application				
Predictors	B	CI	Wald	*p*
Washed my hands more frequently	.191	.63 - 2.34	0.32	.57
Used hand sanitisers *	.588	1.03 - 3.15	4.25	.04
Wore a mask in public voluntarily (before the law was passed)	-.052	.64 - 1.40	0.07	.79
Avoided taking public transport *	.560	1.14 - 2.69	6.51	.01
Stayed home more than usual	-.131	.50 - 1.54	0.21	.65
Avoided crowded places	.245	.73 - 2.24	0.74	.39
Choose outdoor over indoor venues	.408	.99 - 2.30	3.58	.06
Missed or postponed social events	.117	.73 - 1.72	0.29	.59
Changed travel plans	-.099	.61 - 1.36	0.23	.63
Reduced physical contact with others (e.g., avoided shaking hands)	.130	.72 - 1.80	0.31	.58
Avoided visiting hospitals and/or healthcare settings	-.277	.50 - 1.16	1.64	.20
Avoided visiting places where COVID-19 cases were reported	.082	.69 - 1.72	0.12	.73
Kept a distance from people suspected of recent contact with a COVID-19 case	.244	.76 - 2.15	0.84	.36
Kept a distance from people who might have recently travelled to countries with an outbreak	-.149	.51 - 1.45	0.31	.58
Kept a distance from people with flu symptoms	.171	.76 - 1.85	0.57	.45
Relied more on online shopping (e.g., for groceries)	.133	.76 - 1.72	0.41	.52
Stored up more household and/or food supplies than usual	.010	.68 - 1.51	0.00	.96
Took children out of school	.201	.45 - 3.33	0.16	.69
**Model Statistics:**				
Overall percentage of users correctly classified		60.2		
Nagelkerke's R^2		.097		

Table S2. Binary Logistic Regression of the 18 Behavioral Modification with Contact Tracing Application Download as the Dependent Variable
